# Severe Hypocalcemia Leading to Trousseau's Sign

**DOI:** 10.7759/cureus.80401

**Published:** 2025-03-11

**Authors:** Tanique A Burke, Cesar M Almanza, Logan C Hager, Damian Casadesus

**Affiliations:** 1 Internal Medicine, Jackson Memorial Hospital, Miami, USA; 2 Pediatrics, American University of the Caribbean, Sint Maarten, NLD; 3 Neurology, American University of the Caribbean, Sint Maarten, NLD; 4 Surgery, American University of the Caribbean, Sint Maarten, NLD

**Keywords:** chvostek sign, complications post-thyroidectomy, hypocalcemia, thyroidectomy, transaminitis, trousseu's sign

## Abstract

Hypocalcemia is a common complication post-total thyroidectomy. This case report of a young woman with a rare finding on a physical examination highlights the need for patient education, routine laboratory work, and follow-up post-total thyroidectomy. Hypocalcemia is an electrolyte abnormality with numerous causes including chronic kidney disease, vitamin D deficiency, rhabdomyolysis, medications, and hyperventilation. In this report, we discuss the complications and management of hypocalcemia associated with total thyroidectomy. Significant complications include the lifelong need for T4, hypocalcemia, and recurrent and superior laryngeal nerve injuries. The study concludes with the importance of careful primary care follow-ups, medication adherence, and education in postoperative management.

## Introduction

Hypocalcemia is an electrolyte dysfunction characterized by corrected serum total calcium levels < 8.5 mg/dL or uncorrected < 2.12 mmol/L [1]. Hypocalcemia-related disorders are divided into parathyroid hormone (PTH)-mediated and non-PTH-mediated causes, which can be further subdivided into acquired and genetic components. Thyroidectomy is a surgical procedure that involves the removal of all or part of the thyroid gland. This procedure is typically performed to treat conditions such as thyroid cancer, benign thyroid nodules, hyperthyroidism, or goiters. A total thyroidectomy involves the removal of the entire thyroid, whereas a partial thyroidectomy is the removal of a portion of the thyroid. It is important to note that postsurgical hypoparathyroidism (PTH-mediated) is the most common cause of hypocalcemia overall [1].

The diagnosis of postoperative hypocalcemia involves assessing clinical symptoms and confirming the diagnosis with laboratory tests while ruling out other potential causes of hypocalcemia. Clinical symptoms include paresthesias, muscle cramps and spasms, carpopedal spasms, Chvostek's sign, Trousseau's sign, seizures, prolonged QT interval, arrhythmias, and generalized muscle weakness and fatigue. Laboratory values that support postoperative hypocalcemia include reduced serum ionized calcium and PTH levels. Vitamin D levels and renal function tests can be assessed to rule out other causes of hypocalcemia. Hypocalcemic patients often present with tetany, latent tetany, and seizures. Trousseau's sign can be elicited in patients with latent tetany on physical examination with a simple non-invasive maneuver [1].

In this case report, we present a young female patient with significant hypocalcemia complicated by multiple chronic conditions who demonstrated Trousseau's sign on initial physical examination. Hypocalcemia is a relatively common condition observed in hospitalized patients. It is imperative to not only treat hypocalcemia directly, but source determination can significantly alter the modality of treatment.

## Case presentation

A 21-year-old woman with a history of hypoparathyroidism and Graves' disease was admitted to the hospital for evaluation of nausea, vomiting, and numbness in her hands and feet. The patient, who underwent a total thyroidectomy four months prior to admission, was on her eighth admission since the procedure. Her past medical history, recent surgery, and current presentation raised concerns about postsurgical complications and electrolyte imbalance. A carpopedal spasm was elicited while attempting to measure her blood pressure, indicating Trousseau's sign (Video 1).

**Video 1:** Trousseau's sign

The patient's laboratory findings on admission are shown in Table 1.

**Table 1:** Patient's metabolic panel on admission ALT: alanine aminotransferase, AST: aspartate aminotransferase

Measurement	Patient values	Normal values
Calcium	5.7 mg/dL	8.4-10.2 mg/dL
Ionized calcium	0.93 mg/dL	4.2-5.6 mg/dL
25-hydroxyvitamin D	19 ng/mL	20-40 ng/mL
Parathyroid hormone	3 pg/mL	10-60 pg/mL
Thyroid-stimulating hormone	2.92 μU/mL	0.4-4.0 μU/mL
Magnesium	1.2 mEq/L	1.5-2.0 mEq/L
Potassium	5.3 mEq/L	3.5-5.0 mEq/L
Creatinine	0.70 mg/dL	0.6-1.2 mg/dL
Albumin	3.6 g/dL	3.5-5.5 g/dL
AST	106 U/L	10-40 U/L
ALT	74 U/L	12-38 U/L

Elevated liver aspartate aminotransferase (AST) and alanine aminotransferase (ALT) enzymes prompted an abdominal ultrasound, revealing a slightly echogenic appearance of the liver, suggesting hepatosteatosis.

**Figure 1:**
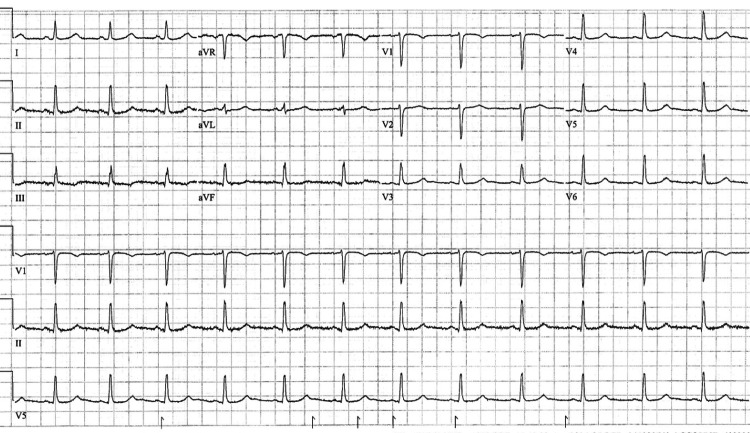
Patient's 12-lead EKG EKG: electrocardiogram

The patient was diagnosed with symptomatic hypocalcemia, a postsurgical complication associated with total thyroidectomy. Upon admission, she began receiving intravenous calcium gluconate, and within the first 24 hours, the paresthesia in her hands and feet subsided. Although she continued to experience nausea at discharge, her vomiting episodes later resolved. To manage her nausea, the patient was prescribed 4 mg of ondansetron. While ondansetron has the potential to cause QT interval prolongation, which is also linked to hypomagnesemia [2], the patient did not exhibit any signs of QT prolongation (Figure 1).

The patient was admitted for 12 days, during which a few laboratory values were evaluated upon discharge. At that time, the patient's total calcium, 25-hydroxyvitamin D, thyroid-stimulating hormone (TSH), magnesium, potassium, creatinine, and albumin levels were all within normal limits. Although her aspartate aminotransferase (AST) and alanine aminotransferase (ALT) showed improvement, they remained elevated. The parathyroid hormone level remained unchanged, and a slight improvement was noted in the ionized calcium level, which still fell below the minimum standard (Table 2).

**Table 2:** Patient's metabolic panel on discharge ALT: alanine aminotransferase, AST: aspartate aminotransferase

Measurement	Patient values	Normal values
Calcium	9.4 mg/dL	8.4-10.2 mg/dL
Ionized calcium	0.15 mg/dL	4.2-5.6 mg/dL
25-hydroxyvitamin D	30 ng/mL	20-40 ng/mL
Parathyroid hormone	3 pg/mL	10-60 pg/mL
Thyroid-stimulating hormone	3.1 μU/mL	0.4-4.0 μU/mL
Magnesium	1.5 mEq/L	1.5-2.0 mEq/L
Potassium	4.7 mEq/L	3.5-5.0 mEq/L
Creatinine	1.00 mg/dL	0.6-1.2 mg/dL
Albumin	4.4 g/dL	3.5-5.5 g/dL
ALT	83 U/L	10-40 U/L
AST	51 U/L	12-38 U/L

The patient was prescribed oral calcitriol 0.75 mcg twice daily and a four-week course of oral cholecalciferol 625 mcg once weekly. Upon discharge, the patient was noted to be relatively asymptomatic, with persistent abnormalities in ionized calcium, parathyroid hormone, AST, and ALT levels. A follow-up with her primary care physician was scheduled to ensure continuity of care and ongoing monitoring of her electrolyte levels and liver function.

## Discussion

Trousseau's sign is a carpopedal spasm induced by inflating a sphygmomanometer cuff 20 mmHg above a patient's systolic blood pressure for three minutes and is commonly observed in hypocalcemic patients [3]. While Trousseau's sign may occasionally appear in healthy individuals, its sensitivity and specificity in hypocalcemic patients are 94% and 99%, respectively [4,5]. Calcium is the most abundant mineral in the body, and low levels of it can cause a plethora of symptoms. Many of the warning signs overlap with other conditions: dry skin, muscle cramps, and tingling in the fingers, toes, and around the mouth.

Post-thyroidectomy patients may be completely asymptomatic or may develop symptoms related to hypocalcemia. Symptoms include tetany, carpopedal spasms, perioral and or digital numbness, seizures, and QT prolongation [6-8]. It has been shown that postoperative vitamin D and calcium supplementation can significantly decrease the risk of transitory hypocalcemia [9] and its associated symptoms.

The patient's elevated liver enzymes and ultrasound findings suggest possible liver dysfunction that could reduce serum protein levels, resulting in less protein-bound calcium and decreased total serum calcium. However, normal albumin levels were confirmed upon admission and discharge, indicating that liver protein production remains adequate. Normal albumin levels lowered concerns about decreased calcium binding and total serum calcium. However, follow-up was recommended to monitor hepatic and calcium status.

## Conclusions

In conclusion, due to the test's high sensitivity and specificity, Trousseau's sign is a common indicator of significant hypocalcemia. Patients with a history of total thyroidectomy should be monitored closely for continued fluctuations in calcium values due to postsurgical primary hypoparathyroidism. Patients should initially undergo calcium checks every 3-6 months. Depending on the outcomes, patients can transition to annual checks. Ultimately, a multidisciplinary approach is necessary to manage their condition correctly, thus reducing symptoms and increasing quality of life.
